# Supplementary material for: End Sequence Analysis Toolkit (ESAT) expands the extractable information from single-cell RNA-seq data
Source: Genome Res. 2016 Oct;26(10):1397–410. doi: 10.1101/gr.207902.116 (PMC5052061; doi:10.1101/gr.207902.116)
Supplement: Supplemental Material [file supp_gr.207902.116_Supp_Materials.tar.gz › ESAT-esat_v0.1/doc/html/classbroad_1_1pda_1_1rnai_1_1_extract_sequence-members.html]

ESAT: Member List


ESAT


- Main Page
- Related Pages
- Packages
- Classes
- Files

- Class List
- Class Index
- Class Hierarchy
- Class Members

All Classes Namespaces Files Functions Variables Enumerator Pages

- broad
- pda
- rnai
- ExtractSequence

broad.pda.rnai.ExtractSequence Member List

This is the complete list of members for broad.pda.rnai.ExtractSequence, including all inherited members.

|  |  |  |
| --- | --- | --- |
| getFirstSequence(String fastaFile) | broad.pda.rnai.ExtractSequence | static |
| getGenomeSequence(GenomicAnnotation annot, String genomeDirectory, boolean repeatMask) | broad.pda.rnai.ExtractSequence | static |
| getSequenceForGene(Collection< Gene > alignments, String genomeDirectory, boolean repeatMask, Map< String, IntervalTree< Alignments >> okRepeats) | broad.pda.rnai.ExtractSequence | static |
| getSequenceForGene(Gene gene, Sequence chrom, boolean repeatMask, Map< String, IntervalTree< Alignments >> okRepeats, boolean stranded) | broad.pda.rnai.ExtractSequence | static |
| getSequenceForGene(Gene gene, Sequence chrom, boolean repeatMask, Map< String, IntervalTree< Alignments >> okRepeats) | broad.pda.rnai.ExtractSequence | static |
| getSequenceForGene(Gene gene, String genomeDirectory, boolean repeatMask) | broad.pda.rnai.ExtractSequence | static |
| getSequenceUnoriented(Alignments align, Sequence chrom, boolean repeatMask, Map< String, IntervalTree< Alignments >> okRepeats) | broad.pda.rnai.ExtractSequence | static |
| getSequenceUnoriented(Alignments align, Sequence chrom, boolean repeatMask) | broad.pda.rnai.ExtractSequence | static |
| getSequenceUnoriented(Alignments align, String genomeDirectory, boolean repeatMask, Map< String, IntervalTree< Alignments >> okRepeats) | broad.pda.rnai.ExtractSequence | static |
| main(String[] args) | broad.pda.rnai.ExtractSequence | static |
| reverseComplement(String seq) | broad.pda.rnai.ExtractSequence | static |
| splitByChr(Collection< Gene > genes) | broad.pda.rnai.ExtractSequence | static |
| writeFasta(String save, Map< String, Collection< Gene >> genesByChr, String genomeDir, boolean repeatMask) | broad.pda.rnai.ExtractSequence | static |


---

Generated on Fri Aug 15 2014 15:11:01 for ESAT by  

 1.8.7
